# NanoMGT: Marker gene typing of low complexity mono-species metagenomic samples using noisy long reads

**DOI:** 10.1093/biomethods/bpae057

**Published:** 2024-08-06

**Authors:** Malte B Hallgren, Philip T L C Clausen, Frank M Aarestrup

**Affiliations:** National Food Institute, Technical University of Denmark, Kemitorvet 204, 2800, Kgs. Lyngby, Denmark; National Food Institute, Technical University of Denmark, Kemitorvet 204, 2800, Kgs. Lyngby, Denmark; National Food Institute, Technical University of Denmark, Kemitorvet 204, 2800, Kgs. Lyngby, Denmark

**Keywords:** metagenomics, marker gene typing, long-read sequencing, Oxford Nanopore Technologies, strain identification, contamination, variant calling, microbial genomics, bioinformatics tools

## Abstract

Rapid advancements in sequencing technologies have led to significant progress in microbial genomics, yet challenges persist in accurately identifying microbial strain diversity in metagenomic samples, especially when working with noisy long-read data from platforms like Oxford Nanopore Technologies (ONT). In this article, we introduce NanoMGT, a tool designed to enhance marker gene typing in low-complexity mono-species samples, leveraging the unique properties of long reads. NanoMGT excels in its ability to accurately identify mutations amidst high error rates, ensuring the reliable detection of multiple strain-specific marker genes. Our tool implements a novel scoring system that rewards mutations co-occurring across different reads and penalizes densely grouped, likely erroneous variants, thereby achieving a good balance between sensitivity and precision. A comparative evaluation of NanoMGT, using a simulated multi-strain sample of seven bacterial species, demonstrated superior performance relative to existing tools and the advantages of using a threshold-based filtering approach to calling minority variants in ONT’s sequencing data. NanoMGT’s potential as a post-binning tool in metagenomic pipelines is particularly notable, enabling researchers to more accurately determine specific alleles and understand strain diversity in microbial communities. Our findings have significant implications for clinical diagnostics, environmental microbiology, and the broader field of genomics. The findings offer a reliable and efficient approach to marker gene typing in complex metagenomic samples.

## Introduction

Modern-day clinical diagnostics are rapidly advancing, driven by innovations in both sequencing technology and analytical solutions. An increasingly popular method for determining bacterial strains is the use of conserved marker gene schemes [1]. Identifying marker genes in pure clinical isolate samples is a well-studied topic, with numerous algorithms already producing high-quality results for both second- and third-generation platforms [2]. However, accurately identifying multiple sets of marker genes in metagenomic samples remains a significant challenge, addressed by only a few tools [3].

Marker gene sets are found in various schemes such as MLST, rMLST, cgMLST, and even mixed sets such as GTDB [4, 5]. These genes are selected for their highly conserved sequence domains and their singular occurrence per genome. If two strains coexist in the same sample, two sets of alleles are expected to be found. Often, a portion of the alleles will be shared among the strains, particularly if they are closely related. Thus, a more accurate representation of allele distribution resembles a Venn diagram, where some alleles are shared and others are unique. The complexity of this distribution increases with the number of strains present. Since the marker genes occur once per sequenced genome, it can be assumed that the depth of coverage for the marker genes is equal to the abundance of the originating genome, assuming randomized whole-genome sequencing [4].

For determining marker gene mutations within a mixed culture or a metagenomic sample, variant calling of the minority variant positions is essential. A minority variant is defined as the presence of an observed nucleotide at a given position within a gene above a specified depth [6]. Different types of variant calling software have been available for more than a decade [7, 8], but most of these were developed specifically for the short-read sequencing platforms available at the time. Newer sequencing technology platforms, such as those offered by Oxford Nanopore Technologies (ONT), produce different types of DNA reads. ONT sequencing platforms are characterized by producing structurally long reads with a high error rate, presenting both challenges and benefits [9]. The longer reads enable a better structural understanding of genome composition as larger read overlaps are available [10]. Additionally, they allow for the coupling of genes or individual mutations, as these might co-occur multiple times within reads originating from the same genome segment [11]. In contrast, shorter sequencing reads with an average length in the range of 100–150 bp, like those produced on most Illumina platforms, cannot capture the structural links between two mutations occurring in non-overlapping reads. Conversely, the high error rate complicates direct gene typing, often leading to false positives and a significant overestimation of identified mutations in the sample [2]. Some studies report a median read accuracy across most organisms of about 90%, while ONT claims that their latest R version 10.4.1 flowcells can achieve accuracies upwards of 99% [12]. Although some tools for variant calling of long reads exist [2], only a few are specifically designed for strain detection in metagenomic samples [3].

Other variant callers that can type long read data, such as Confindr and LongShot [2, 3], adopt different approaches to address sequencing errors in raw reads. Confindr, which is designed to discover contamination in clinical isolates by identifying multiple allele single-nucleotide variants (SNVs) in conserved marker genes, utilizes a proximity pruning scheme similar to tools like CSIPhylogeny [13] and Mintyper [14]. SNVs identified from the alignment’s pile-up are excluded if found in close proximity, as they are considered likely sequencing errors. LongShot uses a different strategy by utilizing a density filter to remove SNVs observed in excess within a moving window. Other tools, such as ONT’s Medaka, are trained on specific error models for error correction. However, pre-trained models often perform well on familiar datasets but may falter on untrained ones.

Other approaches to variant calling for multiple strain detection used by Confindr and LongShot are primarily assembly-based. Tools such as CONCOCT [15] and MetaBAT2 [16] are designed for binning metagenomic assembled genomes based on a series of parameters such as coverage and composition. However, metagenomic assembly is extremely CPU- and memory-intensive, and it has also been shown to produce inaccurate results, including chimeric genomes [17, 18].

In this article, we introduce NanoMGT, a novel tool for accurately calling minority variants in the rMLST marker genes in low-complexity, same-species metagenomic ONT samples. NanoMGT reliably identifies mutations in samples containing multiple strains. Compared to other ONT gene typing pipelines, NanoMGT retains true positives while effectively reducing the noise caused by ONT sequencing errors.

Ideally, NanoMGT should be employed as a post-binning tool in metagenomic pipelines where species separation has been achieved. Specific alleles and the sequence type of their origin can be precisely determined using identified mutations.

## Materials and methods

### Data

To evaluate the variant calling performance of NanoMGT in low-complexity mono-species samples and to identify the error profiles in ONT data, 39 isolates from seven different bacterial species were used to create several multi-strain samples. These isolates were selected from publicly available data, specifically choosing those sequenced on an ONT sequencer using either an r10.3 or r10.4 flow cell. Additionally, most of the samples were chosen for having been base-called using either a super high accuracy (SUP) or high accuracy (HAC) model. Eleven of the samples had unspecified base-calling models in their National Center for Biotechnology Information (NCBI) submissions; however, based on their high Q-scores, these were assumed to be either HAC or SUP samples and were included in the dataset. The sample IDs and their base-calling information can be found in Supplementary Appendix A.

### Identifying the challenges of sequencing errors in ONT data

The sequencing errors from Oxford Nanopore sequencers are primarily deletions, occurring roughly twice the mismatches and insertions rate [9]. In gene typing, incorrect indels are easily addressed by aligning the reads to an appropriate reference. Furthermore, in conserved marker genes, indels are not commonly expected; thus, most of them will likely be attributed to sequencing errors [19].

Mismatch errors are more challenging to reliably identify, as there is no straightforward method to discern whether a mutation in a metagenomic sample results from the presence of a second strain or a sequencing error. Mismatches pose the most significant challenge in terms of accurately identifying multiple sets of marker genes, which are crucial for direct strain detection.

To determine the extent of sequencing errors in ONT data, 39 isolate samples were analyzed for minor variants with a minimum relative positional abundance of 5%. This analysis was performed using NanoMGT with all penalty and reward parameters set to zero and the minimum allele frequency (MAF) parameter set to 0.05. This procedure is equivalent to manually performing a KMA alignment against the highest-scoring reference templates for each rMLST gene, and then calling all minor variants that exceed the set MAF threshold. Each variant was validated for its biological novelty, that is, whether that positional mutation had ever been observed in any rMLST gene. The variants were also checked for occurrence within a proximity of 5 bp to another variant. Additionally, for variants found within 5 nt of one another, their average proximity density was calculated, defined as the average number of additional variants occurring within a 15-bp window on either side of the variant in question. Finally, the number of systematically co-occurring mutations, defined as those having a larger abundance than half of the MAF value when co-occurring with another specific variant, was determined.

### Training and validation datasets

When simulating a multistrain sample, it is crucial to consider the potential contamination of individual isolates. Ideally, in an isolate, there should only be one called variant per position, meaning that no minor variants should be identified. Since benchmarking is based on the variant differences identified between the majority sequences of the mixed isolates, any unaccounted contaminant will introduce additional noise. In a natural multistrain sample, such a contaminant would represent another strain to be identified, and therefore, any typed minor mutations would be considered true positives. However, for the purpose of benchmarking an in-silico dataset, such as the one generated in this study, those minor mutations would be counted as false positives since they do not form part of the expected mutation set.

**Figure 1.**
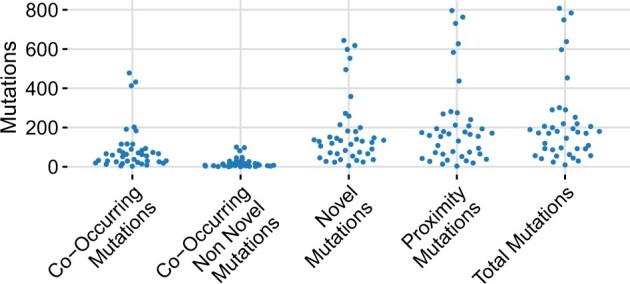
Minority mutations identified with NanoMGT for each bacterial isolate using a MAF value of 0.05 and all other parameters set to zero. About 85.0% of the mutations were found within proximity of another mutation, 77.7% of the mutations were novel and not previously observed in the rMLST database, and 35.2% of the mutations were found to be co-occurring with at least one other mutation. A smaller percentage, an average of 7.8% ranging from 0 to 17.9%, of the mutations were both non-novel and co-occurring with at least one other mutation.

Based on the error profile findings in Supplementary Appendix A and Fig. 1, which are presented and further discussed in the Results and Discussion sections, respectively, the 39 isolates were divided into two subsets: a clean subset (24 isolates) and a contaminated subset (15 isolates). Each of the three subsets and the original combined dataset comprising all 39 isolates were then partitioned into training and validation sets. The partitioning script aims for an 80–20% training–validation ratio but adjusts closer to a 50–50% ratio if the number of isolates is too low. Additionally, if there are insufficient isolates (minimum: 4) within a dataset to produce both training and validation multistrain datasets, then all the isolates were manually selected for either training or validation. Importantly, no isolates were used to simulate both training and validation. Details on the specific allocation of training and validation isolates for all three datasets can be found in Supplementary Appendix A.

The training and validation species with more than three isolates were randomly subsampled into triple-strain samples, and those with two isolates were subsampled into dual-strain samples. In the dual-strain samples, the majority strain ranged from 90 to 99% abundance, and the minority strain from 1 to 10% abundance. In the triple-strain samples, the majority strain ranged from 80 to 98% abundance, while both the minority strains ranged from 1 to 10% abundance. The number of generated strain and abundance combinations depended on the number of isolates but was limited to a maximum of 50 for species that had above five isolates. Each strain composition was simulated to a depth of coverage of 220× using random subsampling with SEQTK (Seed: 100) (https://github.com/lh3/seqtk). Additionally, to test the importance of sequencing depth for minor variant calling, the clean dataset was sampled into depths of 120× and 170×.

The subsampling at different depths was done to determine the impact of sequencing depth on NanoMGT’s variant calling performance. Generally, if strain-specific resolution within a metagenomic sample is desired, it is best to sequence at a very high depth. If a given species is sequenced with a depth of 30× and one of the strains occurs at a 5% abundance, then one would only expect to find 1.5× coverage of that strain’s genome, thus making accurate identification nearly impossible.

The Nanopore P2 solo flow cell can deliver upwards of 580 gigabase pairs of sequencing throughput (https://nanoporetech.com/products/sequence/promethion-2). If a microbial species with a genome of 5M base pairs was found at a 5% abundance in a sample, and the total sample was sequenced with a total throughput of 100 gigabase pairs, that would equate to a 1000× depth of coverage for that species. Therefore, the 120, 170, and 220× coverage simulated in the article is representative of realistic and achievable sequencing depth.

### Typing expected variants

To determine variant positions among the individual bacterial strains, all 39 isolates underwent alignment against the rMLST database using the KMA software [20]. This alignment process generated a consensus sequence for each marker gene within each sample. Subsequently, pairwise alignments of these consensus sequences facilitated the identification of expected mutations utilized for benchmarking purposes.

### NanoMGT pipeline

**Figure 2.**
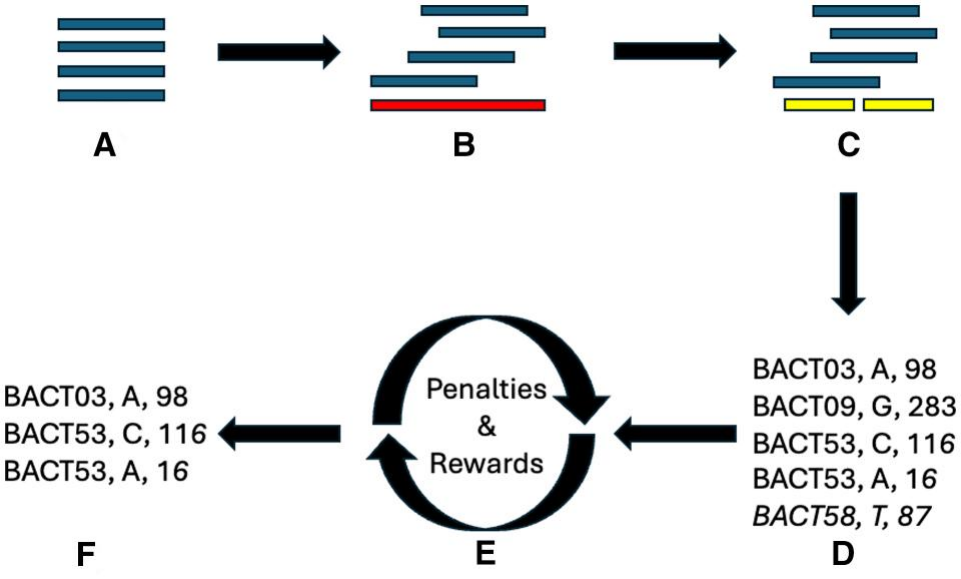
Flowchart of the NanoMGT workflow. (A) Input DNA long reads are trimmed and filtered based on given quality score thresholds. (B) DNA reads are aligned against whole reference genomes to determine species. (C) DNA reads are aligned against the rMLST marker gene set corresponding to the identified species. (D) An initial set of minority variants is identified based solely on the relative abundance at each position. (E) **Algorithm 1** is run until the number of identified mutations converges. (F) The final set of identified minority variants is the output.

NanoMGT is built and evaluated using rMLST bacteria genes. rMLST genes are highly conserved ribosomal sub-unit genes selected in sets of 53 [19]. Any marker gene scheme using the same structural format is compatible and can be used with NanoMGT; however, a larger number of genes will result in longer run times. NanoMGT’s compatibility is only limited by the number of species in the marker gene databases, so over time this number will increase. A visual representation of the entire NanoMGT workflow is shown in Fig. 2.

Initially, input reads are trimmed based on an average read Q-score (default: 14) and mapped using KMA [20] against a homology-reduced version of the NCBI’s RefSeq bacteria database [21] to identify the species present in the sample [22]. The database reduction was performed by indexing with KMA parameters “-ht” and “-hq” set to 0.9, along with the “-and” parameter, which facilitates the removal of highly similar reference sequences to decrease memory requirements. Subsequently, a custom, temporary species-specific marker gene database is generated based on all rMLST alleles identified for that species, effectively narrowing the genomic search space.

The input reads are then aligned with KMA against the species-specific rMLST gene database, and the top allele for each gene is determined [19]. All alignment hits are subsequently realigned against the top allele templates to create a consensus variant map. This map contains the called bases and relative depths for each position in each gene. From the variant map, consensus sequences for each gene can be derived, including potential minority variants with enough depth to pass a given MAF, which is defined as the fractional depth of a mutation at a given position.

The identified minority variants are subject to further filtering to reduce noise. Five rewards and penalties are introduced to penalize or enhance their inclusion threshold:

- **Novel Penalty (np):** Applied when a mutation is biologically novel, that is, not observed in the reference database in any allele. Effect:
  threshold=threshold+MAF×np.
- **Proximity Penalty (pp):** Applied when a mutation occurs within the proximity of nucleotides of another mutation. Effect:
  threshold=threshold+threshold×pp.
- **Density Penalty (dp):** Applied for each additional mutation (M) observed within a proximity of 15 bp. Effect:
  threshold=threshold+MAF×dp×M.
- **Co-occurrence Reward (cor):** Awarded when a mutation consistently co-occurs with the same mutations across multiple reads. Co-occurrence is defined as a mutation occurring with a frequency greater than MAF/2. Effect:
  threshold=threshold−MAF×cor.

The initially typed mutations, surpassing the threshold of MAF×cor, undergo multiple iterations involving the aforementioned rewards and penalties until the count of identified mutations stabilizes. With each iteration, the co-occurrence reward (cor) and the density penalty (dp) are increased by an iteration increase value (ii). This iterative process gradually elevates the threshold for mutations found in clusters, while concurrently lowering it for co-occurring mutations. For true-positive mutations that co-occur within dense mutation regions, these adjustments should roughly balance out, thereby preserving their identification.

The complete threshold convergence algorithm is shown in Algorithm 1

Algorithm 1. SNV Threshold Convergence

1: **Initialization:**

2: MAF←float

3: threshold←MAF×total_positional_depth

4: np←float

5: pp←float

6: dp←float

7: cor←float

8: ii←float

9: original_cor←cor

10: original_dp←dp

11: **procedure**Novel Penalty(*np*)

12:  **if** mutation is novel **then**

13:    threshold←threshold+MAF×np

14:  **end if**

15: **end procedure**

16: **procedure**Proximity Penalty(*pp*)

17:  **if** mutation within 5 bp **then**

18:    threshold←threshold+threshold×pp

19:  **end if**

20: **end procedure**

21: **procedure**Density Penalty(*dp*)

22:  **for** all mutations *M* within 15 bp **do**

23:    threshold←threshold+MAF×dp×M

24:  **end for**

25: **end procedure**

26: **procedure**Co-occurrence Reward(*cor*)

27:  **if** mutation co-occurs **then**

28:    threshold←threshold−MAF×cor

29:  **end if**

30: **end procedure**

31: **procedure**Iterative Adjustment

32:  **while** mutation count not stabilized **do**

33:    Apply Novel Penalty, Proximity Penalty, Density Penalty, Co-occurrence Reward

34:    cor←cor+ii×original_cor

35:    dp←dp+ii×original_dp

36:  **end while**

37: **end procedure**

The user selects the MAF value before running NanoMGT. This value represents the minimum resolution desired to identify minority strains if no rewards and penalties are associated with a given position. Choosing an appropriate value will depend highly on the type and quality of data used, and different MAF values will output different results. Generally, for better data, a lower MAF value may be used, and for data of less quality, users should be content using a higher MAF value, thus not identifying less abundant variants.

### Parameter search

NanoMGT parameters were identified using an iterative grid search. All parameter combinations are initially tested using a wide parameter space, and the best average parameter values across all the simulated multistrain samples are calculated. The initial parameter space chosen was:

- **Novelty penalty interval**: [1, 1.5, 2, 2.5, 3]
- **Proximity penalty interval**: [0.1, 0.2, 0.3, 0.4]
- **Density penalty interval**: [0.01, 0.1, 0.2, 0.3]
- **Iteration increase interval**: [0.01, 0.1, 0.2, 0.3]
- **Co-occurrence reward interval**: [0.1, 0.3, 0.5, 0.7]

Subsequently, four additional iterations of grid searches were conducted with progressively smaller deviations (20%, 15%, 10%, and 5%) from the previously calculated average parameter score. This process continues until a final set of parameter values is determined. Only samples with a simulated depth of 220 were utilized for parameter estimation to limit the computational effort required to search the entire parameter space.

## Results

### NanoMGT parameters

The parameter values identified for NanoMGT using iterative grid search for the clean, contaminated and combined datasets are shown in Tables 1–3.

**Table 1.** Optimized parameters for NanoMGT using the clean dataset.

MAF	cor	ii	pp	np	dp
0.01	0.388	0.156	0.279	3.689	0.234
0.02	0.424	0.129	0.246	3.033	0.228
0.03	0.524	0.161	0.255	2.400	0.074
0.04	0.512	0.055	0.215	2.161	0.160
0.05	0.459	0.0497	0.186	2.009	0.144

**Table 2.** Optimized parameters for NanoMGT using the contaminated dataset.

MAF	cor	ii	pp	np	dp
0.01	0.453	0.179	0.289	4.024	0.213
0.02	0.462	0.196	0.328	3.780	0.167
0.03	0.451	0.102	0.280	3.726	0.182
0.04	0.503	0.1301	0.274	3.719	0.151
0.05	0.513	0.118	0.233	3.450	0.145

**Table 3.** Optimized parameters for NanoMGT using the combined dataset.

MAF	cor	ii	pp	np	dp
0.01	0.502	0.180	0.265	4.022	0.159
0.02	0.483	0.121	0.274	3.732	0.169
0.03	0.453	0.116	0.245	3.235	0.174
0.04	0.528	0.106	0.228	2.811	0.131
0.05	0.536	0.103	0.218	2.793	0.131

The parameter values were fitted using univariate spline interpolation to 500 data points and stored in individual objects representing three models: the combined, clean, and contaminated models. When users of NanoMGT run the software, depending on the parameter model chosen, these data points are loaded and refitted to a spline curve. This process allows the values corresponding to the chosen MAF value to be derived for all five parameters.

### ONT isolate error profiles

The distribution of identified minority variants and their mutation types within the 39 bacterial isolates is illustrated in Fig. 1. The observed number of minority variants per isolate varied widely, ranging from 9 to 808, with an average of 221 variants per isolate. Among these mutations, 85% were found in proximity to another variant, 77.7% were classified as novel, and 35.2% were co-occurring. Additionally, an average of 7.8%, ranging from 0% to 17.9%, of the variants were identified as non-novel and co-occurring. Detailed variant counts, abundances, and species classification for each individual isolate can be found in Supplementary Appendix A.

Table 4 summarizes the minority variant analysis results for individual isolates categorized into three datasets: combined (39 isolates), clean (24 isolates), and contaminated (15 isolates). The table also presents the expected true-positive minor variants based on rMLST consensus sequences from the isolates within these three datasets. The true-positive counts are 2586, 2002, and 4588 mutations for the clean, contaminated, and combined datasets. The abundance of proximity mutations ranges from 6.99 to 7.52%, co-occurring mutations from 18.72 to 19.38%, and novel mutations from 0.54 to 1.20%. For the minor variant profile of the individual isolates, 3295 minor variant positions are observed for the clean dataset, 5380 for contaminated, and 8675 for the combined dataset. The proximity mutations range from 88.40 to 96.23%, the co-occurring mutations from 30.56 to 46.67%, and the novel mutations between 76.42 and 84.65%.

**Table 4.** Minor variants observations for the clean, contaminated, and combined datasets.

Data set	Total variants	Proximity variants	Co-occurring variants	Novel variants
Clean TP	2586	192 (7.42)	484 (18.72)	14 (0.54)
Contaminated TP	2002	140 (6.99)	388 (19.38)	24 (1.20)
Combined TP	4588	332 (7.23)	872 (19.00)	38 (0.83)
Clean Minor SNV	3295	2913 (88.40)	1007 (30.56)	2786 (84.56)
Contaminated Minor SNV	5380	5180 (96.28)	2511 (46.67)	4111 (76.42)
Combined Minor SNV	8675	8093 (93.29)	3518 (40.56)	6897 (79.53)

The true-positive variants were identified by aligning the consensus sequences of the rMLST genes of the 39 isolates pairwise grouped by species. The minor variants in the isolates were identified using only an MAF threshold of 5%. The percentages presented are equal to the abundance of each variant type relative to the total number of minor variants found in the corresponding dataset.

### Analysis with NanoMGT and Confindr

All experiments were carried out using NanoMGT version 1.5.0 and Confindr version 0.8.1. NanoMGT and Confindr were used to analyze the clean, contaminated combined datasets presented in Table 4 with the MAF parameter ranging from 1 to 5% and a minimum of 3 nt required to call a variant. NanoMGT was run with parameters presented in Tables 1–3. Confindr was run with “-b 3,” “-dt Nanopore,” and “-bf” set according to the MAF setting. Both tools used a minimum read quality score of 14 during all analyses. For each result, the precision, recall, and F1-scores were calculated as follows:

$$\begin{array}{c} \text{Precision}=\frac{\text{TP}}{\text{TP}+\text{FP}}\\ \text{Recall}=\frac{\text{TP}}{\text{TP}+\text{FN}}\\ F1-\text{score}=2\cdot \frac{\text{Precision}\times \text{Recall}}{\text{Precision}+\text{Recall}} \end{array}$$

**Figure 3.**
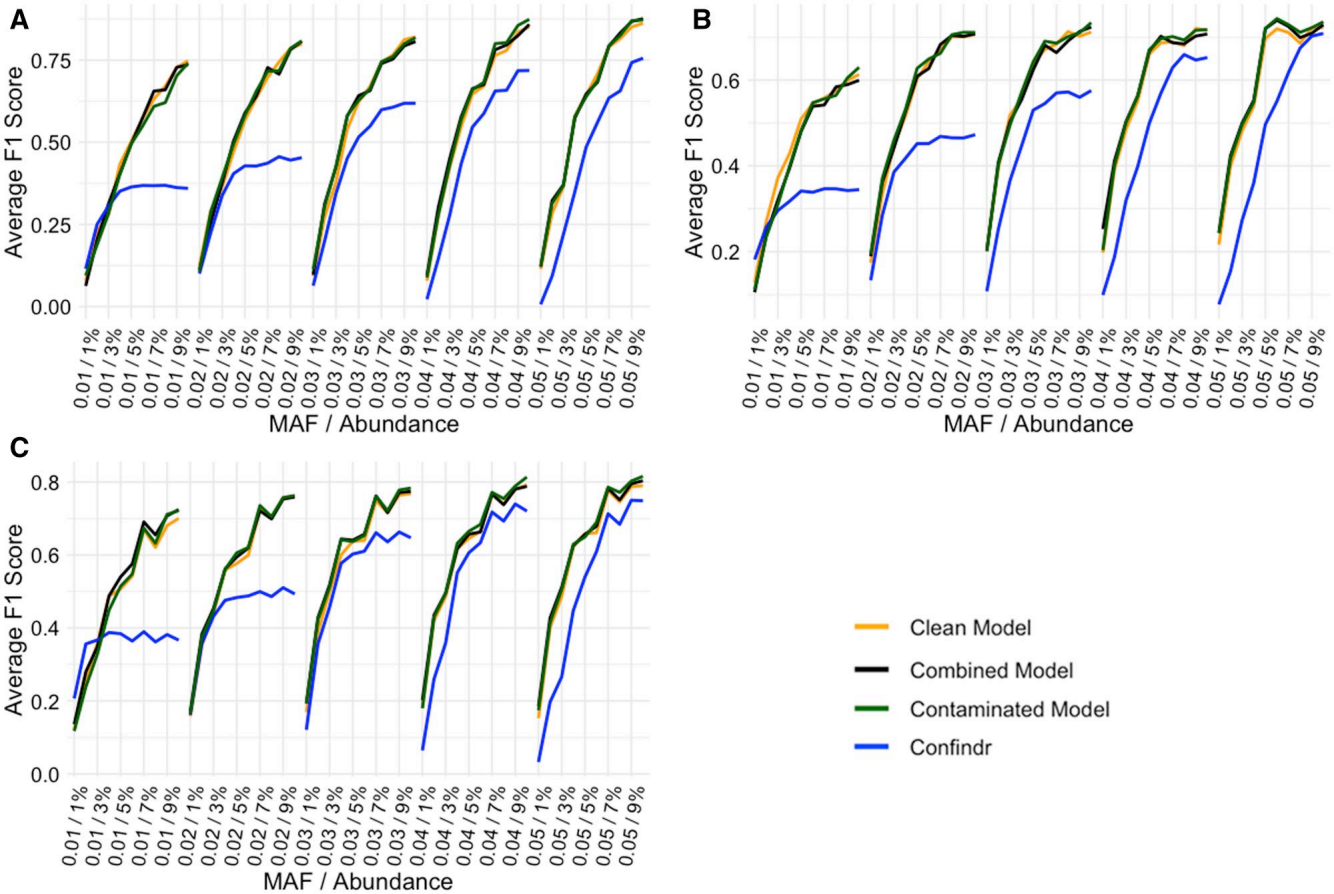
Average F1 performance across the simulated multistrain samples from different datasets using Confindr and NanoMGT run with all three parameter models. The F1-score was calculated for MAF values running from 0.01 to 0.05 (presented as whole percentage integers in the plot) in combination with the abundance of the minority isolates in the multistrain samples running from 1 to 10%. Only every other data point on the *x*-axis is displayed to enhance readability. (A) Clean dataset (24 isolates). (B) Contaminated dataset (15 isolates). (C) Combined dataset (39 isolates).

Figure 3 presents the averaged F1-scores for all three datasets produced by Confindr and NanoMGT using all parameter models. NanoMGT outperforms Confindr across all datasets when the minority abundance is above 2%. The recall and precision scores for the three datasets are detailed in Supplementary Appendix B. It is observed that Confindr performs better in terms of recall for lower MAF values (0.01–0.03), whereas NanoMGT excels at higher MAF values (0.04–0.05). However, regarding precision, NanoMGT outperforms Confindr across all datasets. Most notably, using the clean dataset, NanoMGT achieves upwards of 20% improvement in precision when using an MAF value of 0.05.

### Sample depth related to variant calling performance

Part of the experimental design aimed to evaluate the performance of sample depth. Specifically, the performance of Confindr and NanoMGT, using the clean parameter set, was assessed using the clean dataset at sequencing depths of 120×, 170×, and 220×. For NanoMGT, deeper sequencing depths generally resulted in improved F1 performance across most minimum allele frequency (MAF) and abundance combinations. In contrast, Confindr showed that lower sequencing depths yielded better F1 scores at low MAF thresholds (0.01−0.02), while higher depths were more effective at higher MAF thresholds (0.04−0.05). The detailed recall, precision, and F1 plots for both tools are presented in Supplementary Appendix C.

### Computational requirements

The highest computational requirement for any of the NanoMGT analyses carried out in this article was 10.31 GB RAM and took 4 min 11 s of total wall time when using –threads = 4. The RAM peak originates from the species identification process at the start of the pipeline and is due to the size of the bacterial reference database. 10.31 GB of memory should be manageable by most modern laptops, but in case of a low RAM capacity, the bacterial database can be replaced by a smaller one.

## Discussion

### Error profile of ONT sequencing reads

The error profiles of ONT isolates compared to the expected true positives showed that both novelty and proximity of mutations can be strong indicators of possible sequencing errors. The discrepancy between the identified abundances in the true positives and the isolates often exceeds 80%. Naturally, the amount of proximity mutations will also greatly vary based on the diversity of the samples studied, but this still does not account for the large differences observed. Biological novelty is likely one of the strongest indicators of sequencing errors. It was not impossible to find variant positions that are not yet included in the rMLST gene database, but this occurred only 38 times across all 39 isolates. In contrast to this, the vast majority of minor variants in all three datasets were found to be biologically novel. A plausible explanation could be that most novel variants could impair the gene’s function and are, therefore, not present in any previously sequenced organisms. Systematic or randomized sequencing errors produced by the ONT sequencing platforms would not account for their genetic functional effects. Thus, it intuitively makes sense that a large proportion of noise would be novel.

The larger abundance of co-occurring minor variants identified in the individual isolates compared to the true-positive sets may suggest that certain sequencing errors systematically appear together, potentially complicating the NanoMGT algorithm, which rewards co-occurring mutations. However, considering that over 96.28% of variants were also found in close proximity to one another, a substantial proportion of these co-occurring variants would still be subject to penalties related to both proximity and density.

The method of segregating the 39 isolates into subsets was based on the hypothesis that the combination of non-novelty and co-occurrence likely serves as a strong indicator of a true positive. The isolates were categorized into clean and contaminated subsets to examine the impact of data characteristics on parameter selection. It is crucial to clarify that the naming of the datasets in this study solely reflects the presence of these non-novel co-occurring minor variants. Whether these variants are a product of systematic sequencing errors, the result of contamination, or even the product of index hopping, is not known [23].

### Assessment of identified NanoMGT parameters

Some clear trends occur in the identified parameter values. Most notably, when the MAF value increases, all the penalties (**np**, **pp**, and **dp**) decrease. This is a function of increasing the default threshold (MAF), which by itself filters out a great deal of noise. For the clean dataset, which was selected based on a low number of non-novel and co-occurring minor variants in the isolates, we see the largest reduction in the **np** value as a function of MAF. This happens despite the clean dataset actually having the highest abundance of novel minor mutations occurring in the original 39 isolates. This indicates that the incorrect, novel minor mutations occur at lower abundance in these isolates since removing them takes a lower **np** value.

The iteration increase (**ii**) demonstrates a decreasing trend as a function of the MAF value, suggesting that fewer iterations with smaller **cor** and **dp** increases are required to effectively filter out noise at higher MAF values. The co-occurrence reward (**cor**) slightly increases with the MAF value and generally maintains a high value, indicating a significant benefit in assigning this reward. However, it does not vary substantially across different MAF values. The uniformity in **cor** values across various datasets suggests that the advantages of identifying true positives outweigh the risks of mistakenly assigning the **cor** reward to pairs of false positives.

The identified parameter sets showed that the three datasets selected different values for some parameters at different MAF values. Interestingly, as shown in Fig. 3, there was not a large impact on the performance of any of the datasets despite parameter setting variations. The proximity and novelty penalties are only assigned once, and so they primarily address filtering the bulk of the noise. After that, if any noise remains, the co-occurrence reward and density penalties iteratively increase with the iteration increase factor until two iterations have the same number of variants. Therefore, even if a lower **np** (novelty penalty) or **pp** (proximity penalty) value is selected, there is still a chance that any false positive above the initial threshold will be filtered out in a later iteration.

For optimal performance, the parameters used by NanoMGT might need to be retrained as ONT sequencing technology improves in the future. In less error-prone data, the penalty parameters will likely assume lower values, reducing the risk of excluding true positives.

### Performance of NanoMGT and Confindr

Using all parameter models, NanoMGT outperformed Confindr on the averaged F1 scores for all datasets. Especially at lower MAF thresholds, the performance gap is quite large. The primary driver of this is the novelty penalty that NanoMGT employs. From the expected true-positive mutation set, it is clear that penalizing biological novelty will have only a small effect on a few true positives (0.54–1.20%), whereas it will correctly assign a large penalty to the majority of all observed minor variants (76.42–84.56%). Another important factor is the number of proximity variants. Between 6.99 and 7.42% of the true-positive variants occur within a 5-nt proximity of another variant. Confindr will always filter out all of these variants, whereas NanoMGT can identify them.

Confindr nearly performs as well as NanoMGT on some of the contaminated data for MAF = 0.05 and Abundance = 10%. This aligns with expectations, as this dataset was specifically selected for isolates with the most co-occurring and non-novel minor variants. NanoMGT’s novelty penalty does not penalize these variants, but they do receive the co-occurrence reward, resulting in a lower precision score. This could potentially influence the parameter value during training, especially the **cor** value; however, no significant variation was observed in the parameters identified using any of the three datasets.

NanoMGT’s superior precision performance in Supplementary Appendix B is likely due to its ability to identify false-positive variants accurately. As the parameter selection shows, penalizing variants for being novel is a strong tool for effectively reducing large amounts of noise. This is possible because the rMLST database presents a limited portion of the entire genome for which a large genomic variance has been mapped out. Nonetheless, in future works, it could be interesting to test if building a similar noise reduction function, perhaps based on assessing the translational effect of a given variant, could lead to a similar increase in variant calling precision.

Given the similar performance observed in Fig. 3, the parameter set of the combined model was selected as the default parameters for NanoMGT. This choice provides parameters that generally lie between those of the clean and the contaminated models, ensuring decent performance across all tested datasets.

### Importance of sequencing depth

For NanoMGT, higher F1-scores were observed with higher sequencing depths. However, a few data points for recall and precision indicated that lower depths slightly outperformed higher depths, which could be attributed to randomized sampling variations. It is possible that some mixed samples received more reads from the rMLST region while others accumulated more noise.

Confindr demonstrated improved performance at lower depths for low minor allele frequency (MAF) values. This improvement is due to Confindr’s noise-filtering mechanism, which relies solely on proximity. As sequencing depth increases, more noise is introduced, and such noise may reach sufficient depth to surpass the MAF threshold. As long as these noise signals are not proximal to another variant position, they will be identified as valid. Conversely, at higher MAF values, where most of the noise is filtered out based on abundance, Confindr showed that higher sequencing depths lead to better F1 scores.

### Analysis using other variant callers

It should be noted that the samples analyzed and presented in Fig. 3 were also evaluated using Medaka (https://github.com/nanoporetech/medaka) and LongShot [2]. For both tools, the input reads were aligned against the rMLST genes of the majority abundance samples using Minimap2 [24]. For LongShot, parameters such as –min_alt_frac, –min_alt_count, and –min_cov were adjusted to match those used by NanoMGT and Confindr. Medaka, however, does not accept such parameter inputs and was run directly on each sample without adjustments. Unfortunately, neither tool produced reliable results. Medaka yielded only a few SNVs across all abundance combinations and could not adjust for an MAF threshold, as it does not support such a parameter. LongShot also generated almost no SNVs for the majority of the samples. Even in the optimal experimental condition with a minority variant abundance of 10% and a –min_alt_frac of 0.05, which represents the strongest true-positive signal and the least amount of noise, LongShot identified only a small fraction of the expected variants, thus falling far short of the performance achieved by NanoMGT and Confindr.

Medaka and LongShot, both of which are highly cited and utilized tools, are simply not designed for this specific task. Medaka is built on neural networks trained to refine single-copy elements. Our scenario aims to identify the majority variants that differ from the reference template and the minority variants present at lower abundance. LongShot, on the other hand, could potentially perform comparably for this purpose with some modifications. Specifically, the density filter might need to be adjusted to be less penalizing. In a nanopore metagenomic sample containing multiple strains, one would expect to find multiple error-induced SNVs and a significant number of true-positive SNVs. When combined, an overly aggressive density filter risks excessive trimming, which LongShot will likely do. Additionally, LongShot is primarily designed as a diploid variant caller, where the expected variant ratio within organisms is 50:50, not the 1–10:90–99 ratio simulated in this article.

### Application prospects of NanoMGT

NanoMGT is a tool intended for use downstream in metagenomic analysis pipelines after the raw sequencing data has been binned based on species. Subsequently, NanoMGT can be applied to individual bins of microbial species for which rMLST schemes are available, and based on the typed variants, strain diversity can be determined. Therefore, it should be underscored that NanoMGT is not a stand-alone tool but should rather be used as one of many tools in larger, complex workflows for strain-resolution taxonomic classification within metagenomic samples. Since NanoMGT’s analytical capabilities are limited to the species for which rMLST schemes exist, it will have the highest impact in workflows intended to identify one or more strains associated with infection or disease. This will likely be useful in microbiome diagnostics for clinical patients, but it could also have a significant impact on the analysis of environmental samples from other origins such as water sources or sewage samples.

Another use case for NanoMGT is identifying contamination sources in sequencing data from clinical isolates in quality control pipelines. If the sample has been isolated, running the tool directly with an isolate as input should yield no mutations (naturally accounting for reasonable error rates).

## Conclusion

NanoMGT represents an advancement in the field of microbial genomics, specifically in the typing of marker genes in low-complexity mono-species samples using noisy long reads. This study demonstrated that despite the inherent challenges posed by high error rates in ONT sequencing data, NanoMGT effectively identified minor variants with high precision and recall by employing threshold-based filtering. The tool’s development is a step forward in addressing the complexities of multi-strain identification in sequencing samples, and it offers new opportunities for detailed insights into microbial communities and strain diversity.

## Supplementary Material

bpae057_Supplementary_Data

## Data Availability

All the isolate samples used in this article were public data mined and are accessible at NCBI or ENA by searching the sample IDs from Supplementary Appendix 4. The source code and installation guide for NanoMGT can be found at https://github.com/genomicepidemiology/nanomgt. The scripts used to generate plots, tables, and run analyses for this article can be found in the article_folder on GitHub. For retraining NanoMGT parameters and simulating multistrain samples, follow the instructions in the parameter_training folder. A snapshot of all files in the Github repository for NanoMGT version tag 1.5.0 has been uploaded at https://zenodo.org/records/12709140.
