# Supplementary material for: NanoMGT: Marker gene typing of low complexity mono-species metagenomic samples using noisy long reads
Source: Biol Methods Protoc. 2024 Aug 6;9(1):bpae057. doi: 10.1093/biomethods/bpae057 (PMC11387619; doi:10.1093/biomethods/bpae057)
Supplement: bpae057_Supplementary_Data [file bpae057_supplementary_data.zip › appendix_A.pdf]

Organism	Isolate	Total Mut	Proximity Mut (%)	Novel Mut (%)	Co-occurring Mut (%)	Non-novel Co-occurring Mut (%)	Avg. Prox Density	Basecalling
C. jejuni	SRR26899118	453	437 (96.5%)	358 (79.0%)	183 (40.4%)	38 (8.4%)	28.0	Unknown
C. jejuni	SRR26353490	290	269 (92.8%)	214 (73.8%)	115 (39.7%)	28 (9.7%)	17.3	HAC
C. jejuni	**SRR27638397**	**86**	**64 (74.4%)**	**66 (76.7%)**	**32 (37.2%)**	**7 (8.1%)**	**9.6**	**SUP**
C. jejuni	**SRR27710526**	**170**	**155 (91.2%)**	**135 (79.4%)**	**51 (30.0%)**	**15 (8.8%)**	**10.0**	**SUP**
C. jejuni	SRR27710532	176	165 (93.8%)	140 (79.5%)	63 (35.8%)	17 (9.7%)	11.3	SUP
E. coli	**SRR28370668**	**145**	**131 (90.3%)**	**119 (82.1%)**	**29 (20.0%)**	**7 (4.8%)**	**8.4**	**HAC**
E. coli	SRR24837710	749	731 (97.6%)	598 (79.8%)	413 (55.1%)	81 (10.8%)	41.7	HAC
E. coli	SRR24837712	784	763 (97.3%)	618 (78.8%)	432 (55.1%)	97 (12.4%)	41.6	HAC
E. coli	SRR24834173	808	796 (98.5%)	644 (79.7%)	478 (59.2%)	100 (12.4%)	44.4	HAC
E. coli	**SRR25689478**	**92**	**72 (78.3%)**	**74 (80.4%)**	**25 (27.2%)**	**4 (4.3%)**	**5.6**	**SUP**
E. coli	**SRR26036455**	**96**	**77 (80.2%)**	**71 (74.0%)**	**29 (30.2%)**	**5 (5.2%)**	**6.7**	**SUP**
E. coli	SRR28789463	189	177 (93.7%)	137 (72.5%)	70 (37.0%)	15 (7.9%)	18.8	Unknown
E. coli	SRR28789469	180	171 (95.0%)	130 (72.2%)	73 (40.6%)	18 (10.0%)	17.0	Unknown
E. coli	SRR28800569	170	159 (93.5%)	114 (67.1%)	62 (36.5%)	13 (7.6%)	14.2	Unknown
E. coli	SRR28800580	219	212 (96.8%)	151 (69.0%)	83 (37.9%)	26 (11.9%)	17.3	Unknown
E. coli	**SRR28789754**	**108**	**94 (87.0%)**	**90 (83.3%)**	**59 (54.6%)**	**11 (10.2%)**	**8.8**	**Unknown**
E. coli	**SRR26036458**	**172**	**154 (89.5%)**	**129 (75.0%)**	**25 (14.5%)**	**5 (2.9%)**	**9.4**	**SUP**
k. pneumoniae	**ERR8958810**	**597**	**583 (97.7%)**	**553 (92.6%)**	**191 (32.0%)**	**14 (2.3%)**	**33.5**	**HAC**
k. pneumoniae	**ERR8958737**	**219**	**208 (95.0%)**	**199 (90.9%)**	**66 (30.1%)**	**5 (2.3%)**	**13.2**	**SUP**
k. pneumoniae	**SRR27348733**	**56**	**38 (67.9%)**	**54 (96.4%)**	**14 (25.0%)**	**0 (0.0%)**	**3.2**	**SUP**
k. pneumoniae	**SRR29213739**	**290**	**275 (94.8%)**	**258 (89.0%)**	**116 (40.0%)**	**10 (3.4%)**	**21.9**	**Unknown**
k. pneumoniae	**SRR24833081**	**199**	**174 (87.4%)**	**182 (91.5%)**	**55 (27.6%)**	**3 (1.5%)**	**12.0**	**Unknown**
S. aureus	**SRR28370694**	**9**	**3 (33.3%)**	**5 (55.6%)**	**2 (22.2%)**	**1 (11.1%)**	**0.7**	**SUP**
S. aureus	ERR8958848	180	167 (92.8%)	124 (68.9%)	72 (40.0%)	19 (10.6%)	10.3	HAC
S. aureus	**SRR28370638**	**92**	**74 (80.4%)**	**66 (71.7%)**	**22 (23.9%)**	**5 (5.4%)**	**4.8**	**HAC**
S. aureus	ERR8958843	62	52 (83.9%)	45 (72.6%)	30 (48.4%)	8 (12.9%)	4.8	SUP

Continued on next page

Table 1 continued from previous page

Organism	Isolate	Total Mut	Proximity Mut (%)	Novel Mut (%)	Co-occurring Mut (%)	Non-novel Co-occurring Mut (%)	Avg. Prox Density	Basecalling
S. aureus	SRR25890190	638	627 (98.3%)	495 (77.6%)	202 (31.7%)	47 (7.4%)	32.7	Unknown
S. aureus	**SRR25865495**	**44**	**30 (68.2%)**	**27 (61.4%)**	**16 (36.4%)**	**7 (15.9%)**	**3.0**	**Unknown**
S. enterica	**SRR27755684**	**54**	**33 (61.1%)**	**36 (66.7%)**	**19 (35.2%)**	**5 (9.3%)**	**3.4**	**SUP**
S. enterica	SRR26899146	205	193 (94.1%)	147 (71.7%)	66 (32.2%)	16 (7.8%)	13.0	SUP
S. enterica	SRR27136090	206	194 (94.2%)	145 (70.4%)	93 (45.1%)	30 (14.6%)	12.1	SUP
S. enterica	SRR28399428	252	240 (95.2%)	179 (71.0%)	115 (45.6%)	45 (17.9%)	14.5	SUP
S. enterica	SRR27136088	194	179 (92.3%)	133 (68.6%)	89 (45.9%)	28 (14.4%)	11.8	SUP
S. enterica	**SRR27755678**	**56**	**42 (75.0%)**	**36 (64.3%)**	**18 (32.1%)**	**7 (12.5%)**	**3.1**	**SUP**
L. monocytogenes	**SRR27755667**	**119**	**108 (90.8%)**	**105 (88.2%)**	**37 (31.1%)**	**3 (2.5%)**	**7.7**	**SUP**
L. monocytogenes	**SRR27755674**	**41**	**27 (65.9%)**	**32 (78.0%)**	**12 (29.3%)**	**3 (7.3%)**	**1.9**	**SUP**
L. monocytogenes	**SRR26899103**	**24**	**13 (54.2%)**	**23 (95.8%)**	**4 (16.7%)**	**0 (0.0%)**	**0.9**	**SUP**
L. monocytogenes	**SRR25999202**	**29**	**19 (65.5%)**	**24 (82.8%)**	**8 (27.6%)**	**1 (3.4%)**	**2.5**	**SUP**
P. aeruginosa	**SRR24833086**	**91**	**65 (71.4%)**	**83 (91.2%)**	**29 (31.9%)**	**2 (2.2%)**	**4.5**	**Unknown**
P. aeruginosa	**ERR8958866**	**301**	**281 (93.4%)**	**272 (90.4%)**	**82 (27.2%)**	**10 (3.3%)**	**15.2**	**HAC**

Note: Summary of noise/minority variants in sequencing isolates. Proximity mutations were defined as mutations within five nucleotides of each other; novel mutations were defined as those not previously observed in the rMLST database, and the average proximity density was defined as the number of mutations within a 15-nucleotide window of each mutation. Bold rows denote those isolates used in the clean data set.
