# Supplementary material for: NanoMGT: Marker gene typing of low complexity mono-species metagenomic samples using noisy long reads
Source: Biol Methods Protoc. 2024 Aug 6;9(1):bpae057. doi: 10.1093/biomethods/bpae057 (PMC11387619; doi:10.1093/biomethods/bpae057)
Supplement: bpae057_Supplementary_Data [file bpae057_supplementary_data.zip › appendix_B.pdf]

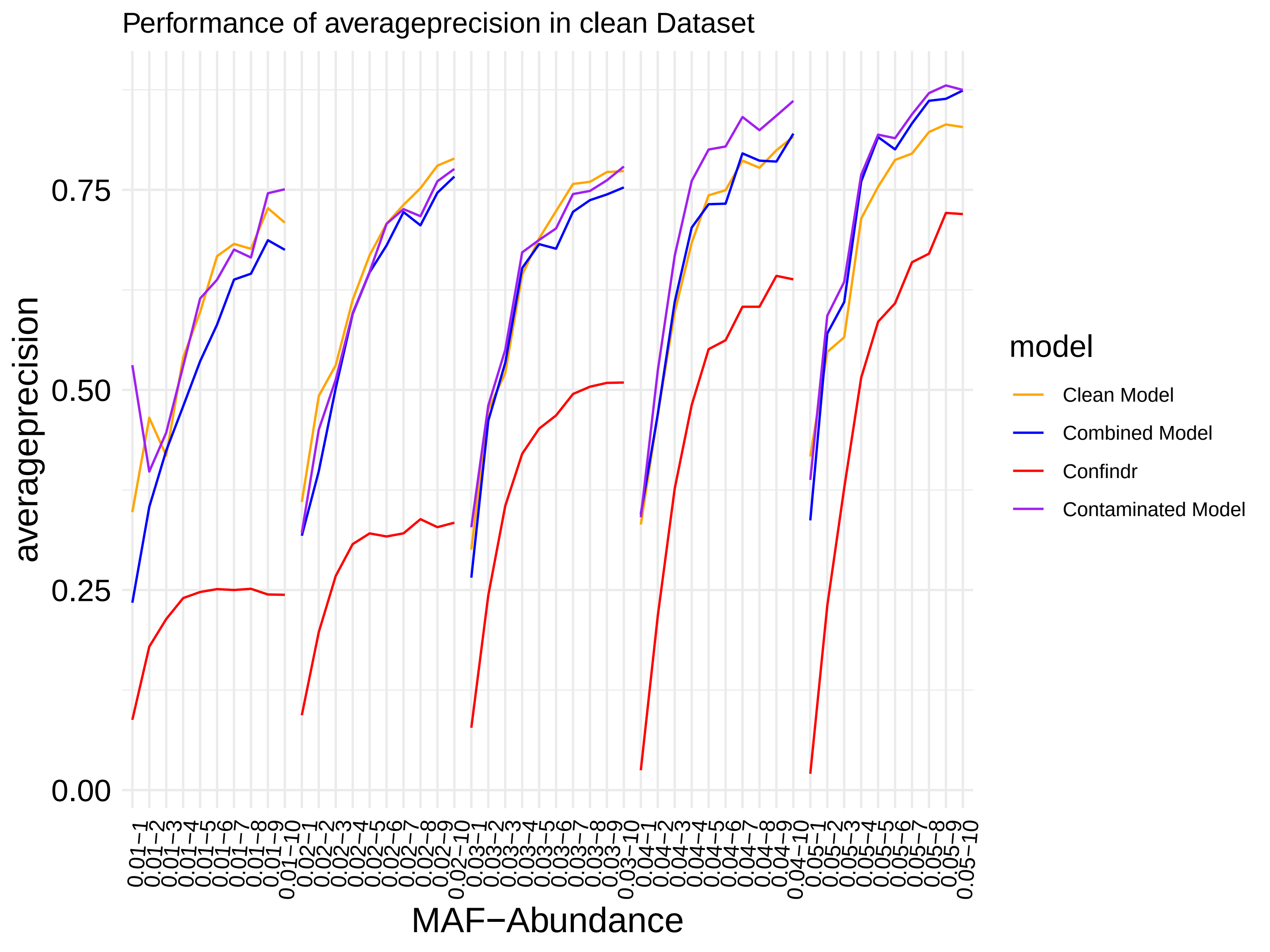

Performance of averageprecision in clean Dataset
averageprecision
0.00
0.25
0.50
0.75
MAF−Abundance
model
Clean Model
Combined Model
Confindr
Contaminated Model

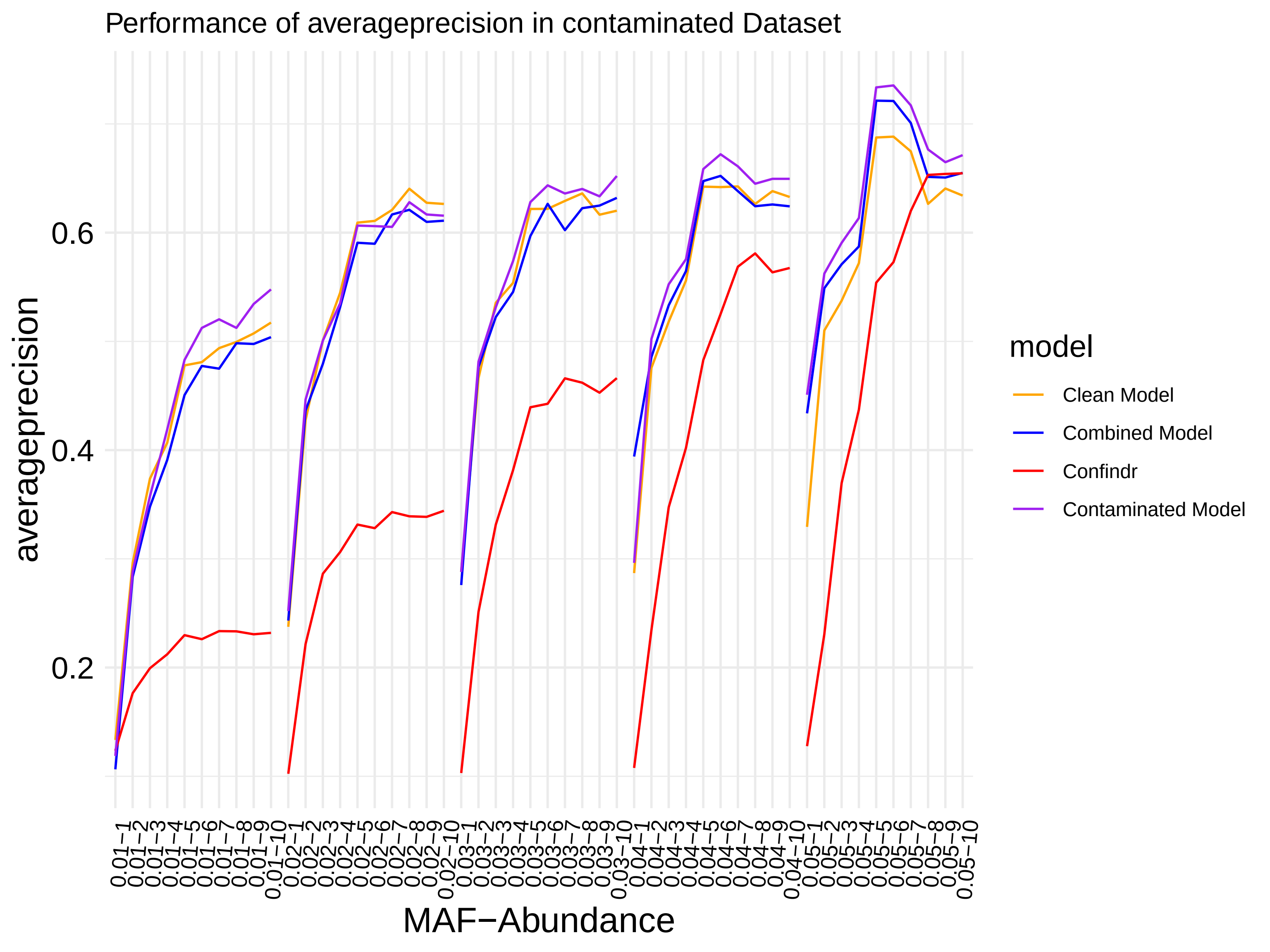

Performance of averageprecision in contaminated Dataset
averageprecision
0.6
0.4
0.2
MAF−Abundance
model
Clean Model
Combined Model
Confindr
Contaminated Model

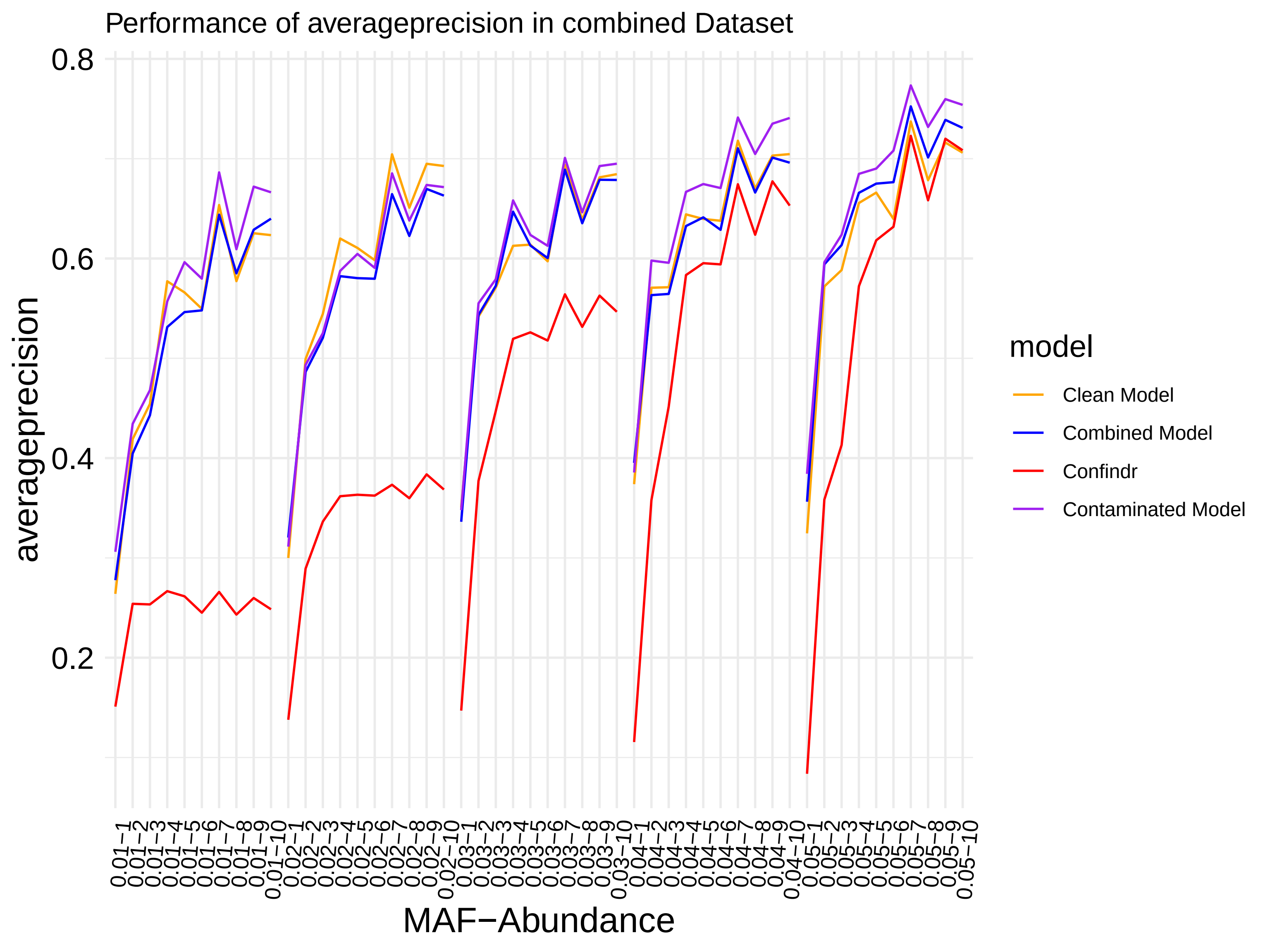

Performance of averageprecision in combined Dataset
averageprecision
0.8
0.6
0.4
0.2
MAF−Abundance
model
Clean Model
Combined Model
Confindr
Contaminated Model

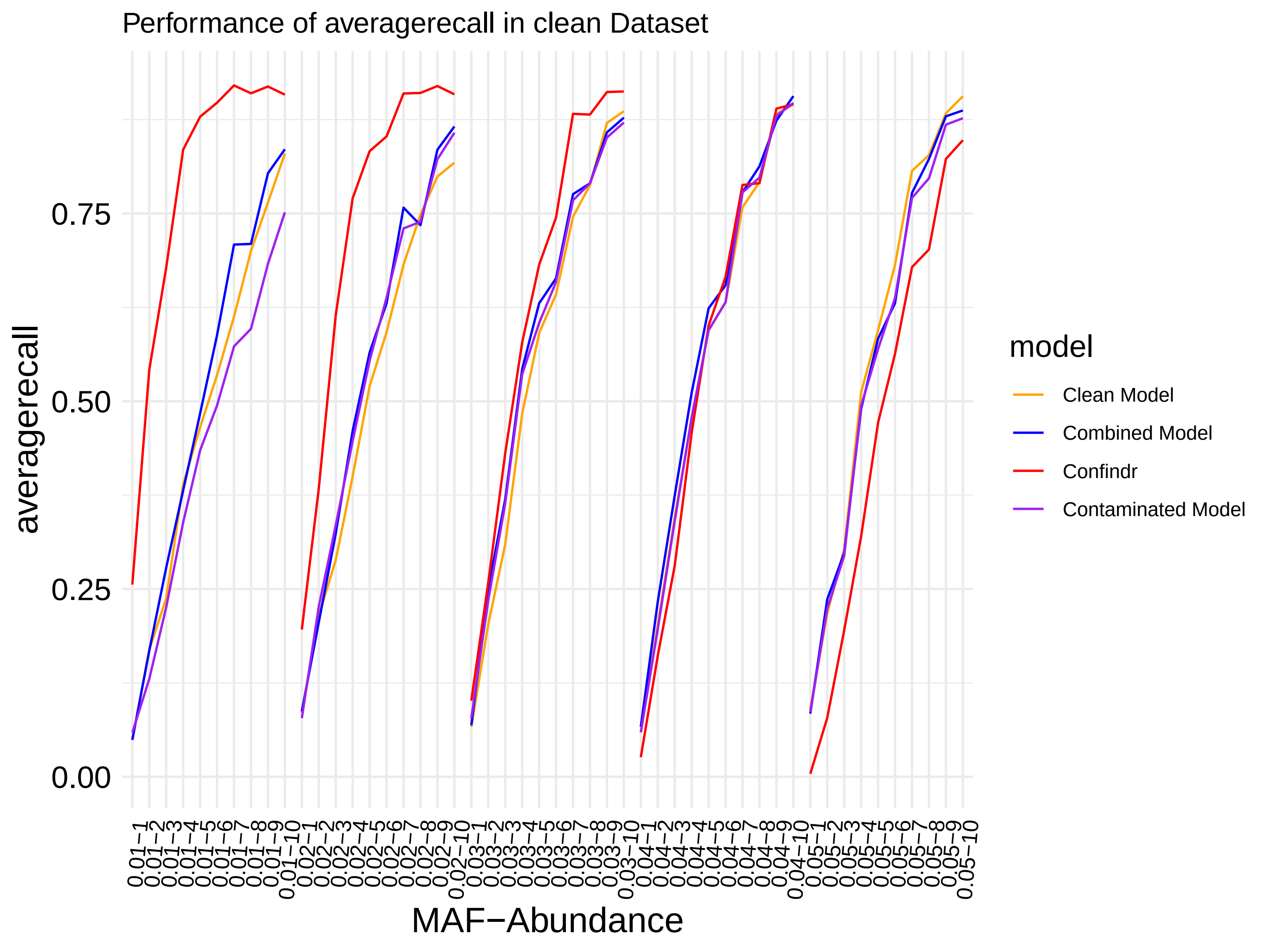

Performance of averagerecall in clean Dataset
averagerecall
0.00
0.25
0.50
0.75
MAF−Abundance
0.01−1
0.01−2
0.01−3
0.01−4
0.01−5
0.01−6
0.01−7
0.01−8
0.01−9
0.01−10
0.02−1
0.02−2
0.02−3
0.02−4
0.02−5
0.02−6
0.02−7
0.02−8
0.02−9
0.02−10
0.03−1
0.03−2
0.03−3
0.03−4
0.03−5
0.03−6
0.03−7
0.03−8
0.03−9
0.03−10
0.04−1
0.04−2
0.04−3
0.04−4
0.04−5
0.04−6
0.04−7
0.04−8
0.04−9
0.04−10
0.05−1
0.05−2
0.05−3
0.05−4
0.05−5
0.05−6
0.05−7
0.05−8
0.05−9
0.05−10
model
Clean Model
Combined Model
Confindr
Contaminated Model

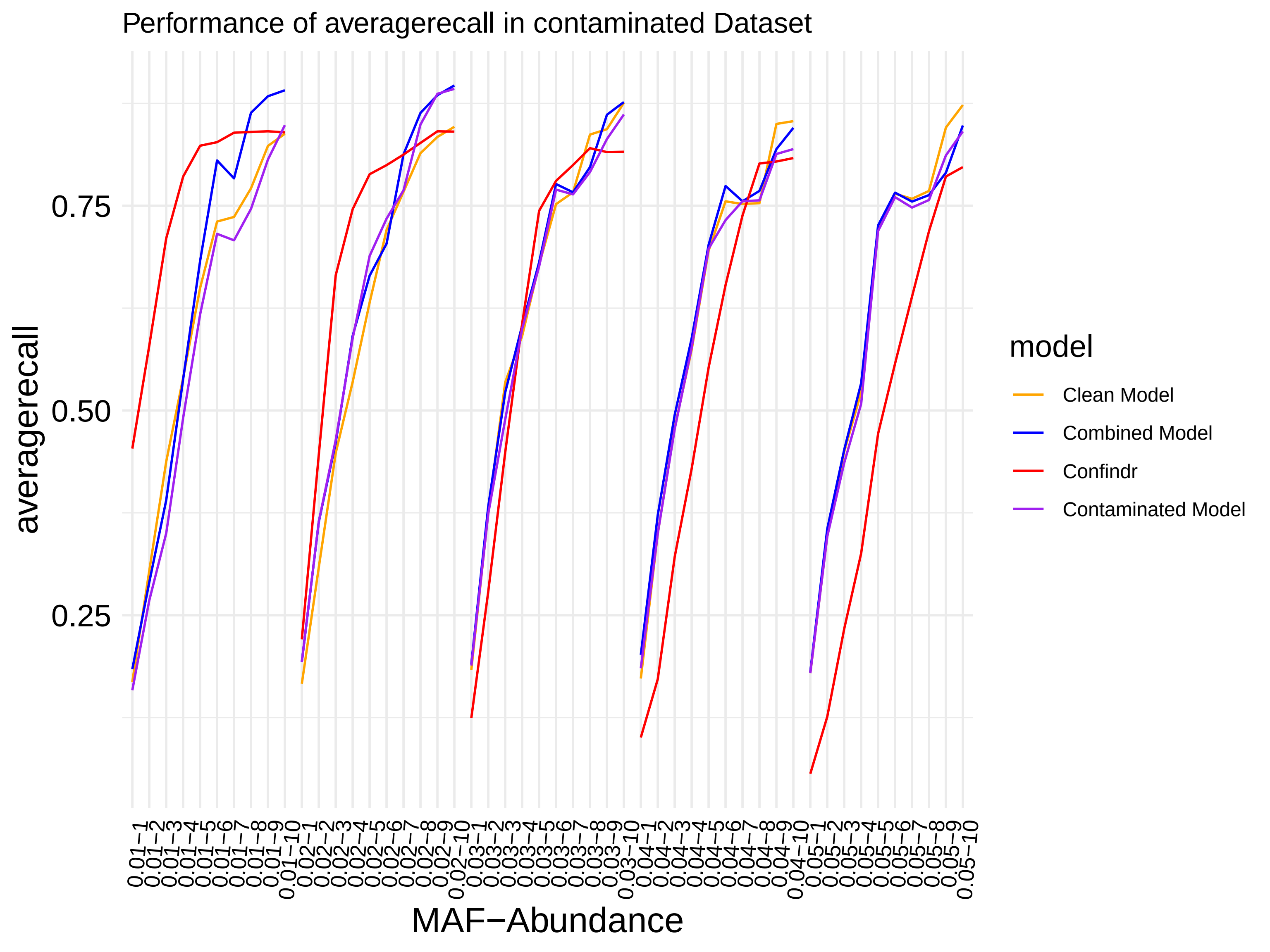
Performance of averagerecall in contaminated Dataset
averagerecall
0.75
0.50
0.25
MAF−Abundance
0.01−1
0.01−2
0.01−3
0.01−4
0.01−5
0.01−6
0.01−7
0.01−8
0.01−9
0.01−10
0.02−1
0.02−2
0.02−3
0.02−4
0.02−5
0.02−6
0.02−7
0.02−8
0.02−9
0.02−10
0.03−1
0.03−2
0.03−3
0.03−4
0.03−5
0.03−6
0.03−7
0.03−8
0.03−9
0.03−10
0.04−1
0.04−2
0.04−3
0.04−4
0.04−5
0.04−6
0.04−7
0.04−8
0.04−9
0.04−10
0.05−1
0.05−2
0.05−3
0.05−4
0.05−5
0.05−6
0.05−7
0.05−8
0.05−9
0.05−10
model
Clean Model
Combined Model
Confindr
Contaminated Model

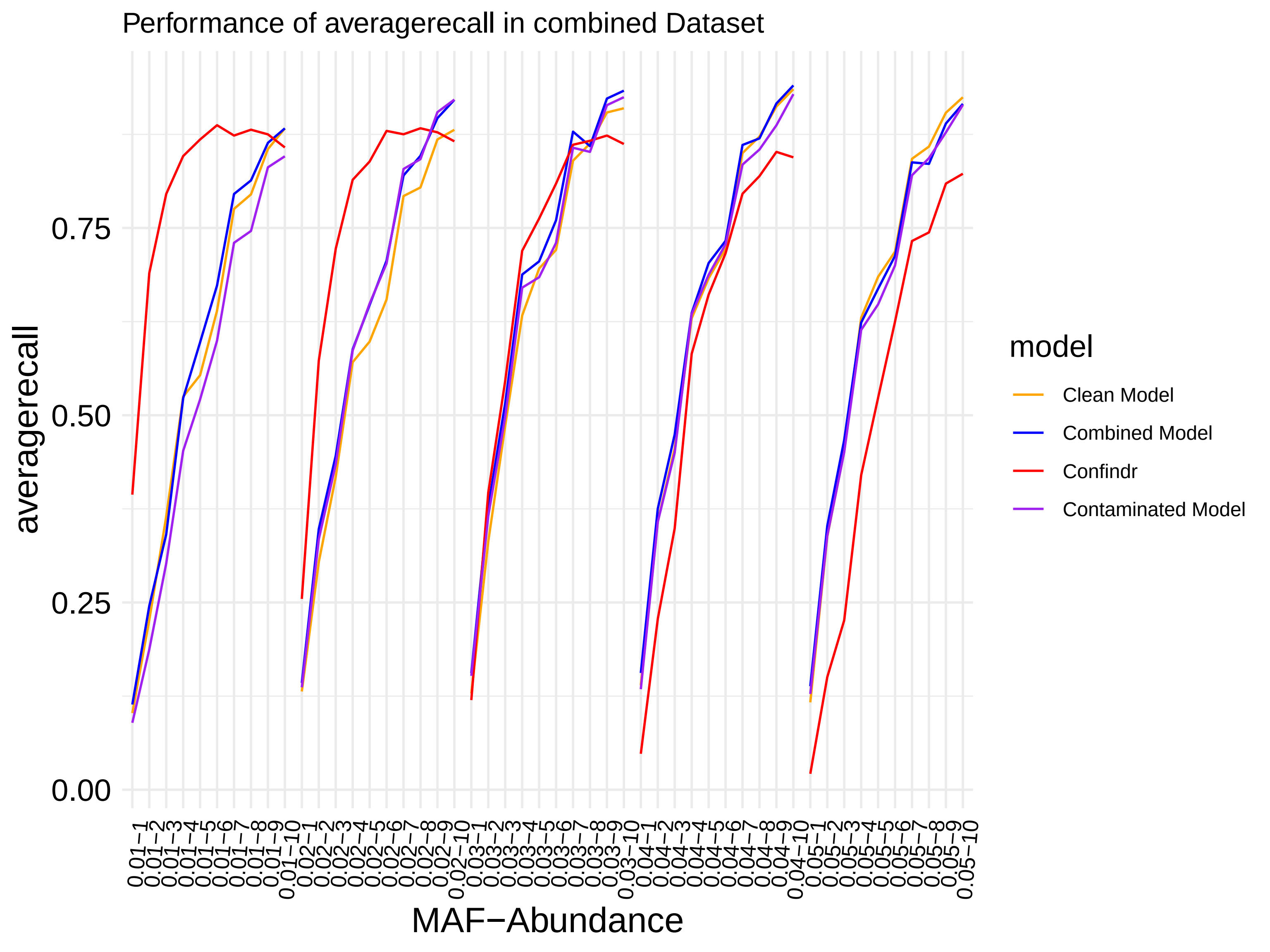

Performance of averagerecall in combined Dataset
averagerecall
0.00
0.25
0.50
0.75
MAF−Abundance
model
Clean Model
Combined Model
Confindr
Contaminated Model
