# Supplementary material for: NanoMGT: Marker gene typing of low complexity mono-species metagenomic samples using noisy long reads
Source: Biol Methods Protoc. 2024 Aug 6;9(1):bpae057. doi: 10.1093/biomethods/bpae057 (PMC11387619; doi:10.1093/biomethods/bpae057)
Supplement: bpae057_Supplementary_Data [file bpae057_supplementary_data.zip › appendix_C.pdf]

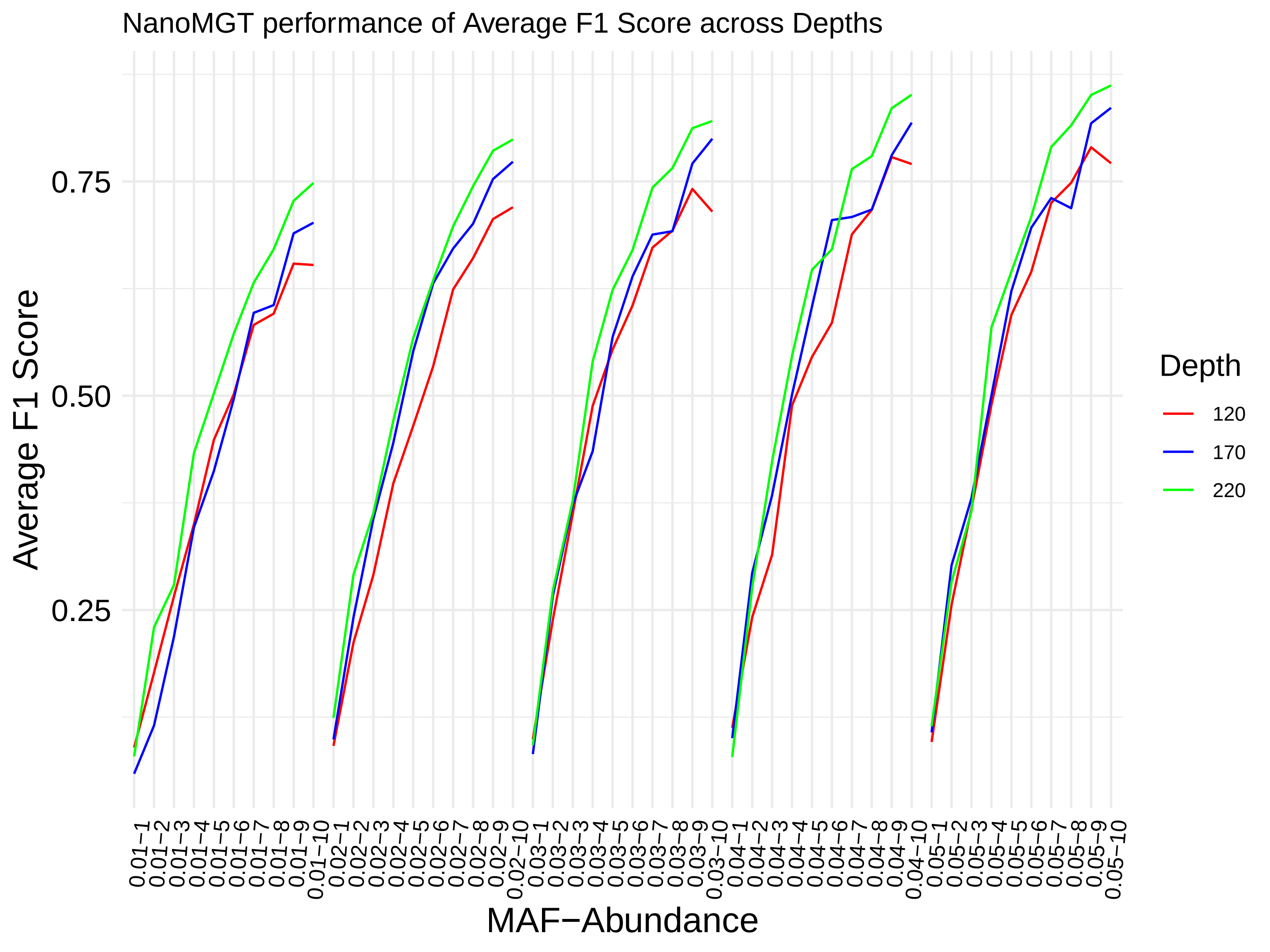

NanoMGT performance of Average F1 Score across Depths
Average F1 Score
0.75
0.50
0.25
MAF−Abundance
Depth
120
170
220
0.01−1
0.01−2
0.01−3
0.01−4
0.01−5
0.01−6
0.01−7
0.01−8
0.01−9
0.01−10
0.02−1
0.02−2
0.02−3
0.02−4
0.02−5
0.02−6
0.02−7
0.02−8
0.02−9
0.02−10
0.03−1
0.03−2
0.03−3
0.03−4
0.03−5
0.03−6
0.03−7
0.03−8
0.03−9
0.03−10
0.04−1
0.04−2
0.04−3
0.04−4
0.04−5
0.04−6
0.04−7
0.04−8
0.04−9
0.04−10
0.05−1
0.05−2
0.05−3
0.05−4
0.05−5
0.05−6
0.05−7
0.05−8
0.05−9
0.05−10

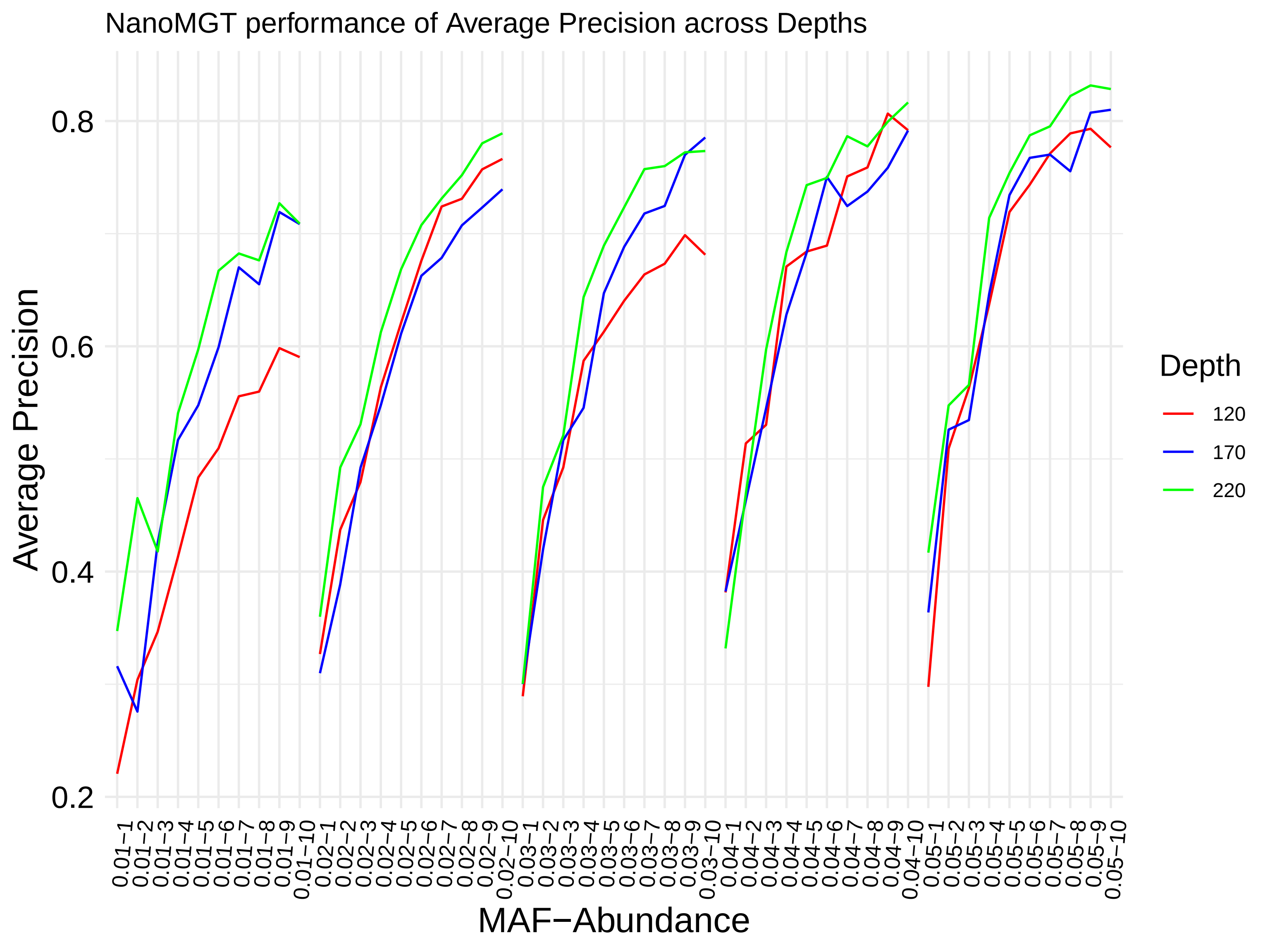

NanoMGT performance of Average Precision across Depths
Average Precision
0.8
0.6
0.4
0.2
MAF−Abundance
Depth
120
170
220
0.01−1
0.01−2
0.01−3
0.01−4
0.01−5
0.01−6
0.01−7
0.01−8
0.01−9
0.01−10
0.02−1
0.02−2
0.02−3
0.02−4
0.02−5
0.02−6
0.02−7
0.02−8
0.02−9
0.02−10
0.03−1
0.03−2
0.03−3
0.03−4
0.03−5
0.03−6
0.03−7
0.03−8
0.03−9
0.03−10
0.04−1
0.04−2
0.04−3
0.04−4
0.04−5
0.04−6
0.04−7
0.04−8
0.04−9
0.04−10
0.05−1
0.05−2
0.05−3
0.05−4
0.05−5
0.05−6
0.05−7
0.05−8
0.05−9
0.05−10

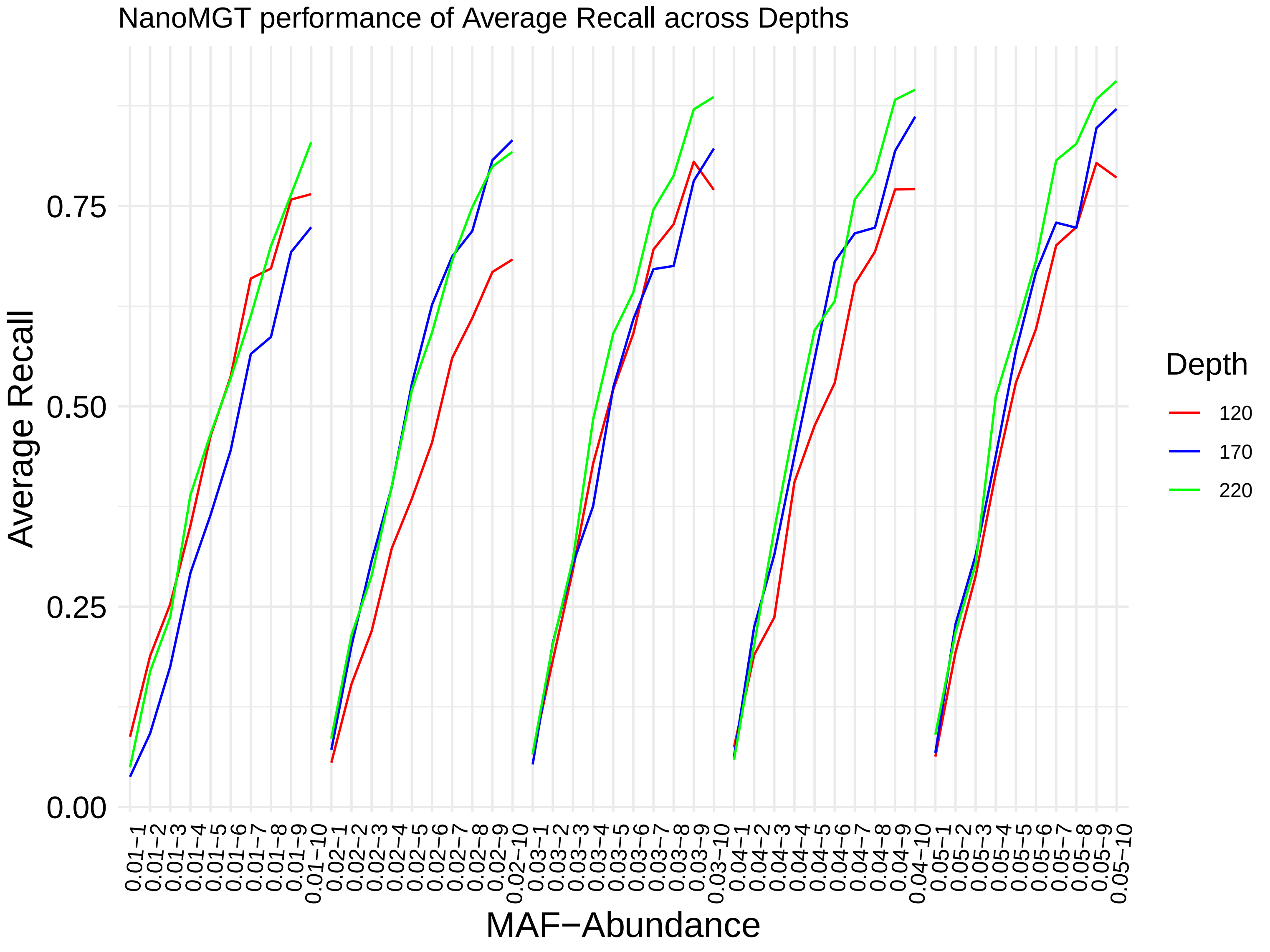

NanoMGT performance of Average Recall across Depths
Average Recall
0.00
0.25
0.50
0.75
MAF−Abundance
0.01−1
0.01−2
0.01−3
0.01−4
0.01−5
0.01−6
0.01−7
0.01−8
0.01−9
0.01−10
0.02−1
0.02−2
0.02−3
0.02−4
0.02−5
0.02−6
0.02−7
0.02−8
0.02−9
0.02−10
0.03−1
0.03−2
0.03−3
0.03−4
0.03−5
0.03−6
0.03−7
0.03−8
0.03−9
0.03−10
0.04−1
0.04−2
0.04−3
0.04−4
0.04−5
0.04−6
0.04−7
0.04−8
0.04−9
0.04−10
0.05−1
0.05−2
0.05−3
0.05−4
0.05−5
0.05−6
0.05−7
0.05−8
0.05−9
0.05−10
Depth
120
170
220

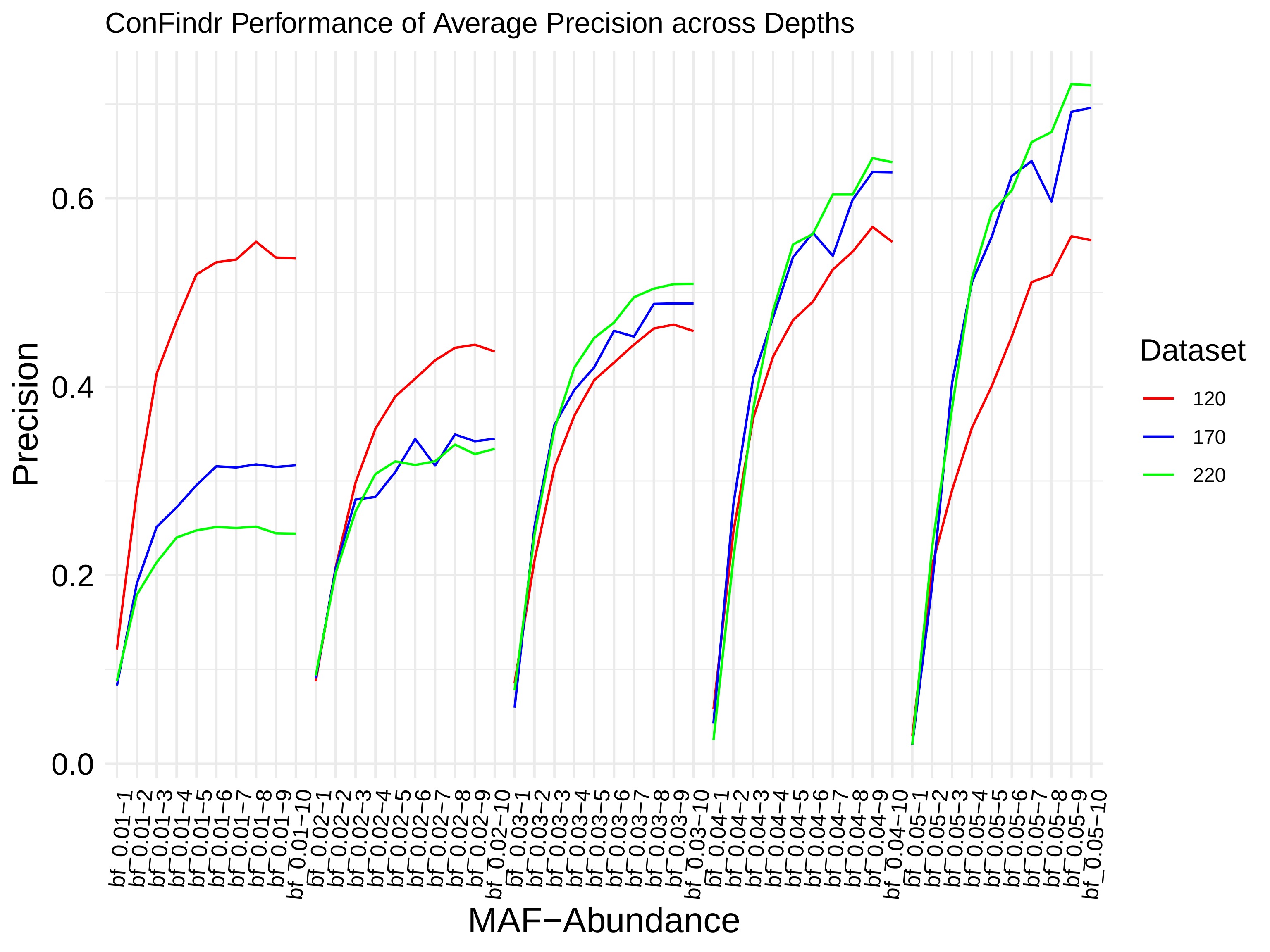

ConFindr Performance of Average Precision across Depths
Precision
0.0
0.2
0.4
0.6
MAF−Abundance
Dataset
120
170
220
bf_0.01−1
bf_0.01−2
bf_0.01−3
bf_0.01−4
bf_0.01−5
bf_0.01−6
bf_0.01−7
bf_0.01−8
bf_0.01−9
bf_0.01−10
bf_0.02−1
bf_0.02−2
bf_0.02−3
bf_0.02−4
bf_0.02−5
bf_0.02−6
bf_0.02−7
bf_0.02−8
bf_0.02−9
bf_0.02−10
bf_0.03−1
bf_0.03−2
bf_0.03−3
bf_0.03−4
bf_0.03−5
bf_0.03−6
bf_0.03−7
bf_0.03−8
bf_0.03−9
bf_0.03−10
bf_0.04−1
bf_0.04−2
bf_0.04−3
bf_0.04−4
bf_0.04−5
bf_0.04−6
bf_0.04−7
bf_0.04−8
bf_0.04−9
bf_0.04−10
bf_0.05−1
bf_0.05−2
bf_0.05−3
bf_0.05−4
bf_0.05−5
bf_0.05−6
bf_0.05−7
bf_0.05−8
bf_0.05−9
bf_0.05−10

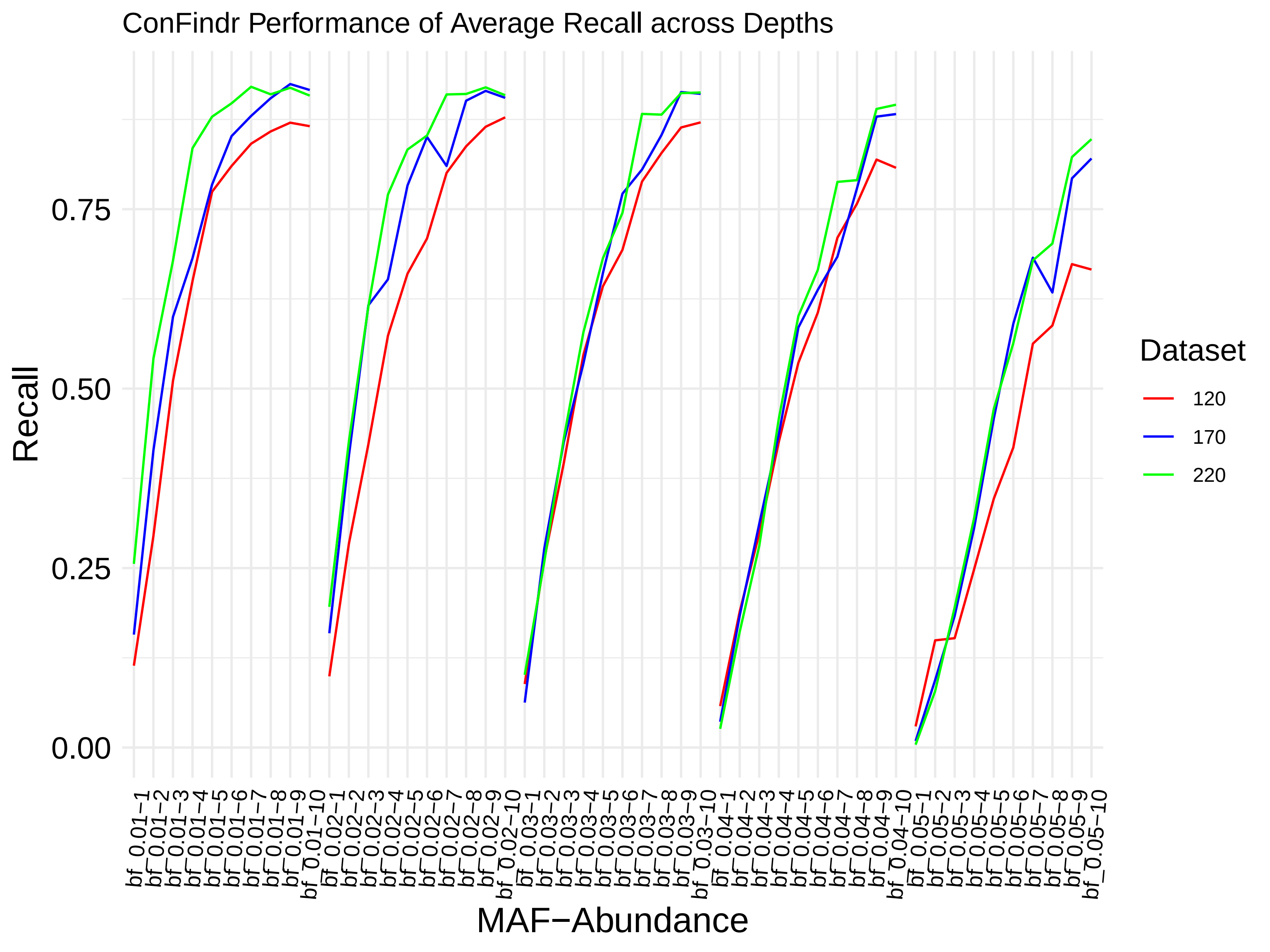

ConFindr Performance of Average Recall across Depths
Recall
0.00
0.25
0.50
0.75
MAF−Abundance
Dataset
120
170
220
bf_0.01−1
bf_0.01−2
bf_0.01−3
bf_0.01−4
bf_0.01−5
bf_0.01−6
bf_0.01−7
bf_0.01−8
bf_0.01−9
bf_0.01−10
bf_0.02−1
bf_0.02−2
bf_0.02−3
bf_0.02−4
bf_0.02−5
bf_0.02−6
bf_0.02−7
bf_0.02−8
bf_0.02−9
bf_0.02−10
bf_0.03−1
bf_0.03−2
bf_0.03−3
bf_0.03−4
bf_0.03−5
bf_0.03−6
bf_0.03−7
bf_0.03−8
bf_0.03−9
bf_0.03−10
bf_0.04−1
bf_0.04−2
bf_0.04−3
bf_0.04−4
bf_0.04−5
bf_0.04−6
bf_0.04−7
bf_0.04−8
bf_0.04−9
bf_0.04−10
bf_0.05−1
bf_0.05−2
bf_0.05−3
bf_0.05−4
bf_0.05−5
bf_0.05−6
bf_0.05−7
bf_0.05−8
bf_0.05−9
bf_0.05−10

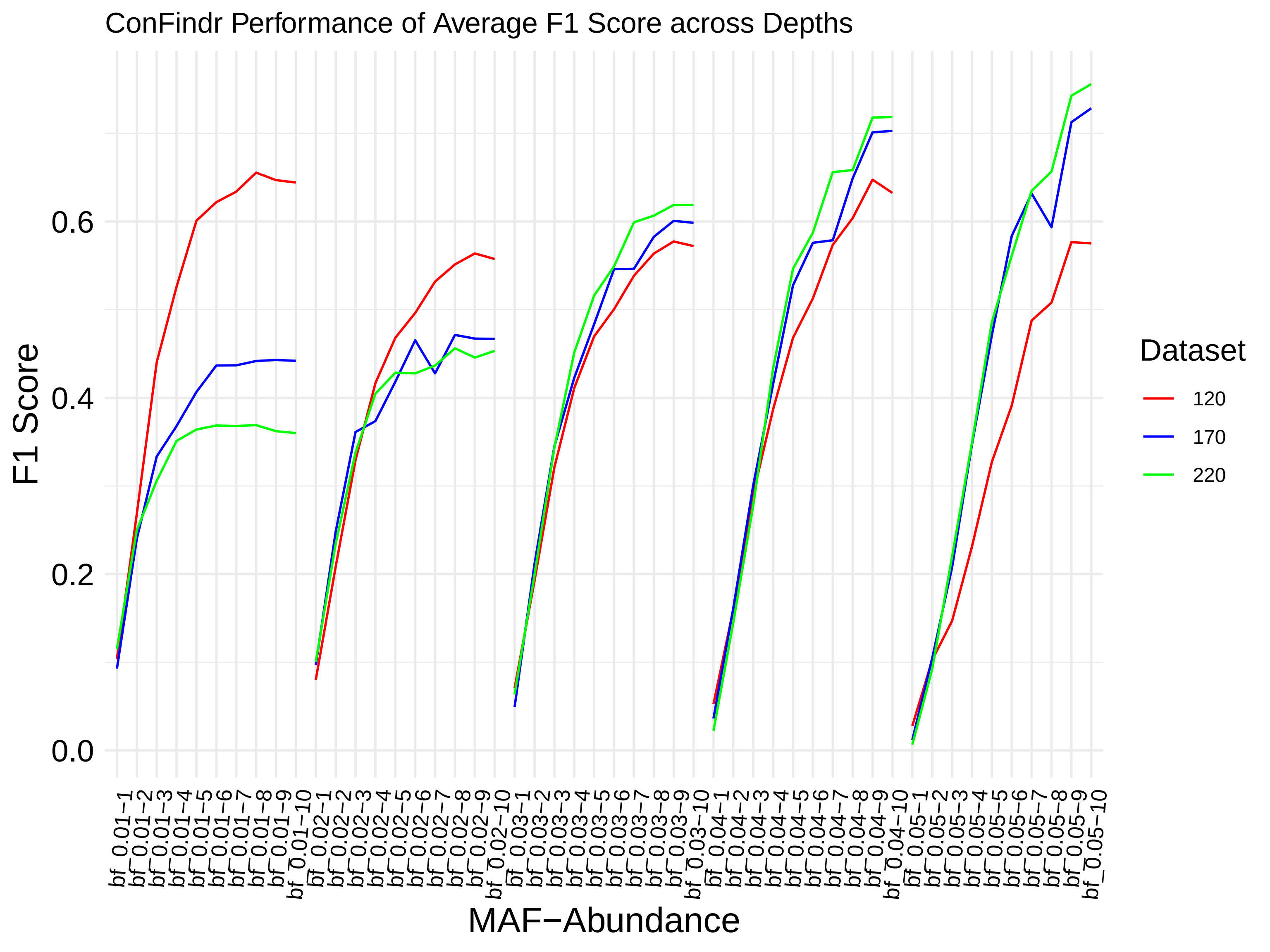

ConFindr Performance of Average F1 Score across Depths
F1 Score
0.0
0.2
0.4
0.6
MAF−Abundance
bf_0.01−1
bf_0.01−2
bf_0.01−3
bf_0.01−4
bf_0.01−5
bf_0.01−6
bf_0.01−7
bf_0.01−8
bf_0.01−9
bf_0.01−10
bf_0.02−1
bf_0.02−2
bf_0.02−3
bf_0.02−4
bf_0.02−5
bf_0.02−6
bf_0.02−7
bf_0.02−8
bf_0.02−9
bf_0.02−10
bf_0.03−1
bf_0.03−2
bf_0.03−3
bf_0.03−4
bf_0.03−5
bf_0.03−6
bf_0.03−7
bf_0.03−8
bf_0.03−9
bf_0.03−10
bf_0.04−1
bf_0.04−2
bf_0.04−3
bf_0.04−4
bf_0.04−5
bf_0.04−6
bf_0.04−7
bf_0.04−8
bf_0.04−9
bf_0.04−10
bf_0.05−1
bf_0.05−2
bf_0.05−3
bf_0.05−4
bf_0.05−5
bf_0.05−6
bf_0.05−7
bf_0.05−8
bf_0.05−9
bf_0.05−10
Dataset
120
170
220
